# ARHGAP11A Promotes the Malignant Progression of Gastric Cancer by Regulating the Stability of Actin Filaments through TPM1

**DOI:** 10.1155/2021/4146910

**Published:** 2021-12-06

**Authors:** Xiaoying Guan, Xiaoli Guan, Junjie Qin, Long Qin, Wengui Shi, Zuoyi Jiao

**Affiliations:** ^1^Pathology Department, Lanzhou University Second Hospital, Lanzhou, Gansu 730030, China; ^2^General Medicine Department, Lanzhou University Second Hospital, Lanzhou, Gansu 730030, China; ^3^Cuiying Biomedical Center, Lanzhou University Second Hospital, Lanzhou, Gansu 730030, China; ^4^The First Department of General Surgery, Lanzhou University Second Hospital, Lanzhou, Gansu 730030, China

## Abstract

The mechanism underlying the poor prognosis of gastric cancer, including its high degree of malignancy, invasion, and metastasis, is extremely complicated. Rho GTPases are involved in the occurrence and development of a variety of malignant tumors. ARHGAP11A, in the Rho GTPase activating protein family, is highly expressed in gastric cancer, but its function and mechanism have not yet been explored. In this study, the effect of ARHGAP11A on the occurrence and development of gastric cancer and the mechanism related to this effect were studied. The expression of ARHGAP11A was increased in gastric cancer cells and tissues, and high ARHGAP11A expression in tissues was related to the degree of tumor differentiation and poor prognosis. Moreover, ARHGAP11A knockout significantly inhibited cell proliferation, cell migration, and invasion in vitro and significantly inhibited the tumorigenic ability of gastric cancer cells in nude mice in vivo. Further studies revealed that ARHGAP11A promotes the malignant progression of gastric cancer cells by interacting with TPM1 to affect cell migration and invasion and the stability of actin filaments. These results suggest that ARHGAP11A plays an important role in gastric cancer and may become a useful prognostic biomarker and therapeutic target for gastric cancer patients.

## 1. Introduction

Cell migration is a complex and dynamic process involving the continuous remodeling of cellular structure [1]. Migration is an important feature of gastric cancer cell spreading during metastasis. To successfully metastasize, cancer cells must cross the barriers constituted by blood vessels, tissues, and the extracellular matrix. Cancer cells can migrate collectively or individually, and cells can change their migration behavior dynamically and reversibly [2]. Studies have found that Rho GTPases play an important role in the occurrence of malignant tumors, regulating a variety of biological processes, such as cytoskeletal reorganization, cell motility, and cell cycle progression [3]. As molecular switches, Rho GTPases cycle between the active GTP-bound state and the inactive GDP-bound state. This process is regulated by guanine nucleotide exchange factors (GEFs) and Rho GTPase-activating protein (RhoGAP) [4].

To study the main mechanisms involved in the malignant progression of gastric cancer, we performed gene expression profiling on 16 pairs of gastric cancer and adjacent tissues. The results showed that ARHGAP11A, in the Rho GTPases-activating protein family, exhibited significantly increased expression in gastric cancer tissues. ARHGAP11A is a nonspecific RhoGAP that regulates the conversion of Rho GTPases to the GDP-bound form. We found that through bioinformatic analysis ARHGAP11A is highly expressed in a variety of cancer tissues, and Fan's research also confirmed this [5]. Another researchers identified 17 highly expressed key genes in gastric cancer tissues by weighted gene coexpression network analysis, and ARHGAP11A is one of them. Studies have shown that ARHGAP11A plays a crucial role in the maintenance of gastric cancer stem cells [6]. ARHGAP11A promotes colon cancer cell invasion and is the main regulator of cancer cell motility [7]. In basal-like breast cancer cells, ARHGAP11A has oncogenic rather than tumor-suppressive effects [8] and is an ideal target for the treatment of invasive tumors. ARHGAP11A is related to the infiltration of immune cells (CD8+ T cells, CD4+ T cells, macrophages, and dendritic cells) in gastric cancer and may be an important regulator of immune infiltrating cells and a valuable prognostic marker [5]. However, the role of ARHGAP11A in the occurrence and development of gastric cancer, as well as whether ARHGAP11A affects the proliferation and metastasis of gastric cancer cells, whether interacting proteins are involved in its role, and whether specific molecular signaling pathways are involved in its regulation, needs to be further studied.

In this study, we found that ARHGAP11A is the most highly expressed gene of the Rho GTPase-activating protein family in gastric cancer. Its function of regulating the stability of actin microfilaments and promoting the proliferation, invasion, and migration of gastric cancer cells depends on TPM1, thus promoting the malignant progression of gastric cancer. The important role of ARHGAP11A in gastric cancer provides us with a new potential therapeutic target for the study of gastric cancer.

## 2. Material and Methods

### 2.1. Tissue Specimens

A total of 432 pairs of gastric cancer and paracancerous tissues were obtained between 2016 and 2019 after written informed consent was obtained. Postoperative pathological sections were diagnosed by experienced pathologists. None of the patients received chemotherapy or radiotherapy before surgery, and the procedure was approved by the Ethics Committee of Lanzhou University Second Hospital.

### 2.2. Cell Lines and Cell Culture Conditions

GES-1 human gastric mucosal epithelial cells and HGC-27, MKN-45, NCI-N87, and AGS human gastric cancer cell lines were obtained from the Institute of Basic Medical Sciences, Chinese Academy of Medical Sciences (Beijing, China). HEK293T cells were obtained from the ATCC; all cells were cultured in a cell incubator at 37°C in 5% CO_2_.

### 2.3. Plasmid Construction and Cell Transfection

First, two sgRNA targets were designed and synthesized, the sequences of sgRNA were KO1: GGCAATGTACGCTTAGCATT, KO2: TGGTTTCCACCAATGAGTAC, and the sgRNA was amplified and ligated to the Lenti-CRISPR vector (at the BsmBI site) using the Gibson Assembly method. The plasmid was transformed into competent cells and extracted with a Tiangen Plasmid Extraction Kit (Tiangen, China). The lentiviral core plasmid Lenti-CRISPR Puro-ARHGAP11A and two packaging plasmids psPAX2 and pMD2G were cotransfected into HEK293T cells using Lipofectamine 2000 (Invitrogen, USA). After 48 hours, the supernatant was collected and ultracentrifuged for infection of gastric cancer cells. The sequence of sgRNA for TPM1 is GCCCGTAAGCTGGTCATCATT; the specific protocol used for plasmid construction is the same as that described above.

Regarding construction of the ARHGAP11A overexpression plasmid, to increase the expression of ARHGAP11A, two pairs of primers were used to construct the stable transfection plasmid Lenti-CMV-Flag-ARHGAP11-puro and the transient transfection plasmids PRK5-Flag-ARHGAP11A and PRK5-HA-ARHGAP11A; the related primers sequences are shown in Supplemental Table S1. The primers sequences of truncation mutants of ARHGAP11A are shown in Supplemental Table S2.

### 2.4. Immunohistochemistry

A total of 432 pairs of paraffin-embedded gastric cancer and adjacent tissues were made into tissue chips. The sections were dewaxed and hydrated via routine methods, and endogenous peroxidase activity was blocked with 3% hydrogen peroxide. The sections were subjected to antigen repair in a pressure cooker containing citrate buffer and incubated with immunohistochemical blocking solution at room temperature for 30 minutes. After adding the primary antibody, the sections were placed in a refrigerator overnight at 4°C. Primary antibody ARHGAP11A (1 : 300, Affinity, USA) was used. Next, the sections were developed with diaminobenzidine (DAB), and the coloration reaction was terminated by washing with distilled water. After staining with hematoxylin staining solution, 1% hydrochloric acid-alcohol (75% ethanol: hydrochloric acid = 100 : 1) was used for differentiation. The sections were dehydrated in an alcohol gradient and cleared with xylene, and neutral gum was then used to seal the slides. Scoring was performed by experienced senior pathologists according to the positive staining intensity of tumors and the percentage of staining: 0, 0%; 1, <25%, 2, 25%–75%; 3, 75%–100%. The final result was expressed as the H-score value, H-Score = ∑(*i* × *Pi*), where *i* is the staining intensity and Pi is the percentage of stained cells.

### 2.5. Quantitative Real-Time PCR Analysis

Quantitative real-time PCR was performed as described previously [9]. TRIzol reagent (TaKaRa, Japan) was used for total RNA extraction, and a TaKaRa kit RR047A (TaKaRa, Japan) was used for reverse transcription according to the protocol. A TB Green™ Fast qPCR Mix Kit (TaKaRa, Japan) was used to perform real-time PCR analysis in a Roche LightCycler 96 real-time quantitative PCR instrument (Roche, Switzerland). The reaction conditions for the standard two-step PCR amplification procedure were as follows:

Stage1: predenaturation at 95°C 30 s, 1 cycle; Stage 2: amplification at 95°C for 5 s and 60°C for 20 s, 40 cycles; Stage 3: melting curve analysis at 95°C for 0 s, 65°C for 15 s, and 95°C for 0 s. GAPDH was used as the reference gene. The relative fold changes in the mRNA levels were calculated using the 2^−ΔΔCT^ method. The sequences of the primers used are shown in Table 1. The primers were synthesized by Xi'an Qingke Zexi Biotechnology Co., Ltd.

### 2.6. Western Blot Analysis

Cellular protein was extracted with RIPA lysis buffer (Solarbio, China), the protein concentration was measured with a BCA protein quantitative kit (Solarbio, China), and the absorbance value was measured at 562 nm in a microplate reader. 25 micrograms of total protein was added to each well. Proteins were separated by 8%–12% sodium dodecyl sulfate- (SDS-) polyacrylamide gel electrophoresis (PAGE) and transferred to a PVDF membrane. After blocking with 5% skimmed milk for 1 hour, the membrane was incubated overnight at 4°C with primary antibodies specific for the following proteins: ARHGAP11A (1 : 1000, Affinity, USA), TPM1 (1 : 500, Abcam, UK), GAPDH (1 : 1000, Proteintech, USA), and *β*-actin (1 : 1000, Proteintech, USA). The membrane was washed with TBST on a shaker and was then incubated with the corresponding secondary antibody (1 : 10000, Proteintech, USA). The immunoreactive protein bands were visualized using an enhanced chemiluminescence kit (Xinsaimei, China).

### 2.7. Detection of Cell Proliferation with the High Content Analysis System

The cell density was adjusted to 1 × 10^4^ cells/ml after conventional trypsin digestion. 200 *μ*L cell suspension was added to each well of a 96-well plate so that the number of target cells was 2000. Each group of cells to be tested was established with 5 replicate wells. The plate was put into the High Content Analysis System (PerkinElmer, USA) and prepared for counting. The parameters of the High Content Analysis System were set after the cells had adhered to the wall; the cells were counted every 3 h and observed continuously for 120 h. Statistical analysis was conducted according to the number of cells counted at each time point.

### 2.8. EdU Detection of Cell Proliferation

Cells (5 × 10^5^ cells/ml) were plated in a 48-well plate, labeled with EdU (Solarbio, China) according to the protocol, fixed with 4% paraformaldehyde, washed with PBS, and permeabilized with 0.3% TritonX-100. The reaction solution was then prepared according to the instructions, 0.1 ml Click reaction solution was added to each well, and the cells were incubated at room temperature for 30 minutes and stained with 1x Hoechst 33342 solution at room temperature for 30 minutes. The High Content Analysis System was used to perform fluorescence detection at the corresponding wavelength.

### 2.9. Detection of Colony Formation

Cells (200 cells/well) were seeded in 35 mm dishes, cultured in 2 ml of DMEM (Gibco, USA) containing 10% FBS for two weeks, fixed with 1 ml of 4% neutral formaldehyde for 10 minutes, washed with PBS 3 times, and then stained with 1 ml of 1% crystal violet, photographed, and counted with Image-Pro Plus software.

### 2.10. Cell Invasion and Migration Assays

The cell migration ability was evaluated by a wound-healing assay. Cells (5.0 × 10^5^ cells/well) were seeded in a 6-well plate. When the cells were confluent, a 100 *μ*L sterile pipette tip was used to make three vertical scratches in each well. PBS buffer was gently added from the sidewall and the cells that detached from the wound were removed by washing. Images were taken and sampled at 0 h, 24 h, and 48 h, respectively, and the scratch lengths in each group at each time point were measured with ImageJ software.

A Transwell invasion assay was used to evaluate the cell invasion ability. The membrane in each chamber was coated with Matrigel matrix (BD Biosciences, USA), and the cell suspension was resuspended in serum-free medium to adjust the cell density to 1 × 10^5^ cells/ml. 200 *μ*L aliquot of the cell suspension was added to each upper chamber, and 600 *μ*L of complete culture medium containing 10% serum was added to each lower chamber. Culture was continued at 37°C for 48 h. A clean cotton swab was used to gently wipe away the cells on the upper surface of the chamber membrane. Then, the remaining cells were fixed with 4% neutral formaldehyde for 10 minutes and stained with 0.1% crystal violet at room temperature for 1 hour. The membranes were visualized and imaged under a microscope. Cells were counted with Image-Pro Plus software.

### 2.11. Immunofluorescence

Cells were seeded in a small glass dish, and the culture medium was discarded the next day. Cells were washed three times with PBS, fixed with 4% cold paraformaldehyde for 20 minutes, permeabilized with 0.1% Triton X-100 for 5 minutes, and blocked with immunohistochemical blocking solution for 30 minutes. The prepared Rhodamine-Phalloidin solution (1 : 300, Solarbio, China) and prepared DAPI solution (1 : 500, Solarbio, China) were added at a volume of 300 *μ*L, and the cells were placed in a wet box for staining in a 37°C incubator for 2 hours. The High Content Analysis System was used to detect and visualize fluorescence and acquire images in the corresponding channel. The Skeleton and Strahler analysis plugins in Fiji ImageJ software were used to extract and analyze the structure of cytoskeletal actin filaments. The Skeleton plugin can extract the cytoskeleton visualized by immunofluorescence staining to obtain a linear cytoskeleton structure. The Strahler analysis plugin can display the cytoskeleton in different colors according to the complexity of its connections and can measure a series of parameters, such as the number of cytoskeletal branches, the length of the branches, and the number of connection points, to evaluate the degree of cytoskeletal variation. The website for the cytoskeleton analysis process is http://imagej.net/Analyze; the website for the Strahler cytoskeleton analysis plug-in is http://imagej.net/Strahler_Analysis#Root_Detection.

### 2.12. Nude Mouse Tumorigenicity Assay

MKN45 cells in the control group and ARHGAP11A knockout group were prepared into cell suspension, and the cells were counted. Twenty 4-week-old male nude mice were randomly divided into two groups, and the mice in each group were injected subcutaneously with 5 × 10^6^ cells. Tumor formation of nude mice after injection was observed regularly every day. The length and width of the tumors were measured with a Vernier caliper, the tumor volume was calculated with equation V = *L* × W^2^/2 mm^3^, and a tumor growth curve was drawn. At week 5, nude mice were anesthetized with 1% sodium pentobarbital, necropsied, and photographed. The experiment was approved by the Medical Ethics Committee of Lanzhou University Second Hospital. All animal experiments were carried out in accordance with Guidelines for Ethical Review of Laboratory Animal Welfare of China.

### 2.13. Comprehensive IP Assays to Screen Interacting Proteins of ARHGAP11A

Cells in each group were collected and lysed with lysis buffer containing 1% protease inhibitor (1 ml: 1 M Tris-HCl (pH-7.4) 50 *μ*l, 1 M NaCl 150 *μ*l, 0.5 M EDTA 2 *μ*l, 10% TritonX-100 20 *μ*l, and H_2_O 778 *μ*l). After centrifugation at 13000 rpm, 50 *μ*l of the cell lysate supernatant was taken as the WCL. The remaining cell lysate was added to 20 *μ*l Flag gel beads and incubated with rotation at 4°C for 2 h. The gel beads were washed with 1 ml lysis buffer, centrifuged at 5000 rpm at 4°C for 3 min, and washed again 3 times. 50 microliters of elution buffer (0.1 M Glycine, adjusted with HCl to pH 3.5) was added. After incubation for 5 min and centrifugation at 8000 rpm for 3 min, the above elution steps were repeated for 4 times, a total of 200 *μ*l elution solution was obtained, and 24 *μ*l of neutralization buffer (0.5 M Tris-HCl (pH 7.4), 1.5 M NaCl) was then added. A small amount of the sample was added to 5x SDS loading buffer, and protein was denatured by boiling for 10 min. After centrifugation at 2500 rpm and 4°C for 1 min, Western blot was performed. The remaining samples were frozen at -80°C and sent to Beijing Haiteng Biotechnology Co., Ltd., for mass spectrometry analysis.

### 2.14. Coimmunoprecipitation

The experiment was performed as described previously [10]. The cell processing procedure was the same as that described above. Cells were eluted with 50 *μ*l of elusion Buffer and centrifuged at 8000 rpm for 3 min; the supernatant was then transferred into a new EP tube, and then 6 *μ*l of neutralization Buffer (0.5 M Tris-HCl (pH 7.4), 1.5 M NaCl) was added. Then, 5x SDS loading buffer was added to the samples and boiled for 10 min. After centrifugation at 2500 rpm for 1 min at 4°C, Western blot was performed.

### 2.15. Statistical Analysis

SPSS 23.0 (IBM, USA) was used to perform statistical analyses. The chi-square test was used to compare differences between two or more groups. A *t*-test was used to analyze differences between the mean values of two groups, and one-way ANOVA was used to analyze differences among more than two groups. The data are expressed as percentages and mean ± standard deviation. *p* < 0.05 are considered significant.

## 3. Results

### 3.1. ARHGAP11A Is Overexpressed in Gastric Cancer and Is Associated with Poor Outcomes in Gastric Cancer

**Figure 1:**
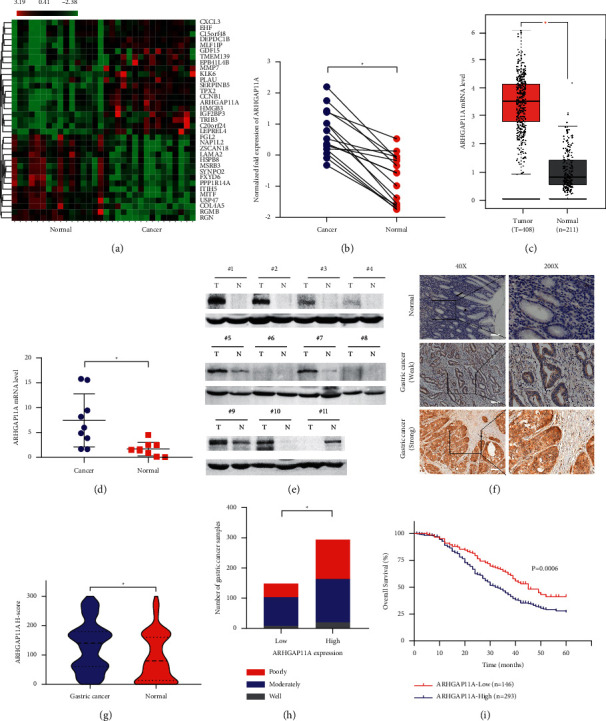
Expression of ARHGAP11A in gastric cancer patients and its relationship with prognosis. (a) Bioinformatic analysis showed that ARHGAP11A was highly expressed in various types of cancer tissues. (b) ARHGAP11A expression was increased in gastric cancer tissues compared with normal tissues. (c) Gene chip analysis of the expression profile showed that ARHGAP11A was highly expressed in gastric cancer tissues. (d) qRT-PCR was used to detect the expression of ARHGAP11A mRNA in gastric cancer and normal tissues. (e) Western blot was performed to detect the expression of the ARHGAP11A protein in gastric cancer and normal tissues. (f) Immunohistochemistry method detects weak and strong positive expression of ARHGAP11A in gastric cancer tissues. The scale bar indicates 200 *μ*m. (g) Quantitative analysis of ARHGAP11A expression in gastric cancer and corresponding adjacent tissues after immunohistochemical staining by comparison of H scores. (h) Kaplan-Meier survival analysis was performed on 432 patients with gastric cancer. (i) Comparison of the number of gastric cancer patients with different differentiation levels in the high and low ARHGAP11A expression groups; the difference was statistically significant. ^*∗*^*p* < 0.05.

First, we performed gene expression profile analysis on 16 pairs of gastric cancer and normal tissues and found that ARHGAP11A, a member of the RhoGAP family, showed a signficantly increased expression (Figure 1(a)). The mRNA expression level of ARHGAP11A was higher in gastric cancer tissues than in adjacent normal tissues (Figure 1(b)).

The expression of ARHGAP11A in various types of cancer tissues was further analyzed in the GEPIA2 database (http://gepia.cancer-pku.cn/), and the mRNA levels of ARHGAP11A in 408 human gastric cancer samples and 211 normal gastric tissues were detected. The results showed that ARHGAP11A was highly expressed in lung cancer, breast cancer, pancreatic cancer, esophageal cancer, gastric cancer, and other types of cancers (Supplementary Figure S1) and that the mRNA expression level of ARHGAP11A was higher in gastric cancer tissues than in adjacent normal tissues (Figure 1(c)). Upregulation of ARHGAP11A was further verified by qRT-PCR analysis of gastric cancer/normal tissues (Figure 1(d)), Western blot analysis of 11 pairs of tissues (Figure 1(e)), and immunohistochemical analysis of 432 pairs of samples in a gastric cancer TMA (Figures 1(f) and 1(g)); the results were consistent with our previous conclusion obtained by bioinformatic analysis. To further explore whether the expression level of ARHGAP11A is correlated with the prognosis of gastric cancer patients, Kaplan-Meier survival analysis was performed on the 432 patients. The prognosis of the ARHGAP11A high-expression group (*n* = 293) was worse than that of the ARHGAP11A low-expression group (*n* = 146) (*p*=0.0006, Figure 1(i)). This result shows that high expression of ARHGAP11A is related to the poor prognosis of gastric cancer patients.

The gastric cancer specimens were further studied according to different clinicopathological characteristics, and the correlations between the expression level of ARHGAP11A and the clinicopathological characteristics of different patients were explored by comparing the differences of ARHGAP11A expression levels between the different groups. The results showed that the expression level of ARHGAP11A was closely related to tumor differentiation (*p* < 0.05, Figure 1(h) and Table 1) but was not significantly correlated with age, sex, clinical stage, TNM stage, or other clinicopathological characteristics of the patients (*p* > 0.05; Table 2).

### 3.2. ARHGAP11A Promoted Gastric Cancer Cell Proliferation In Vitro and In Vivo

**Figure 2:**
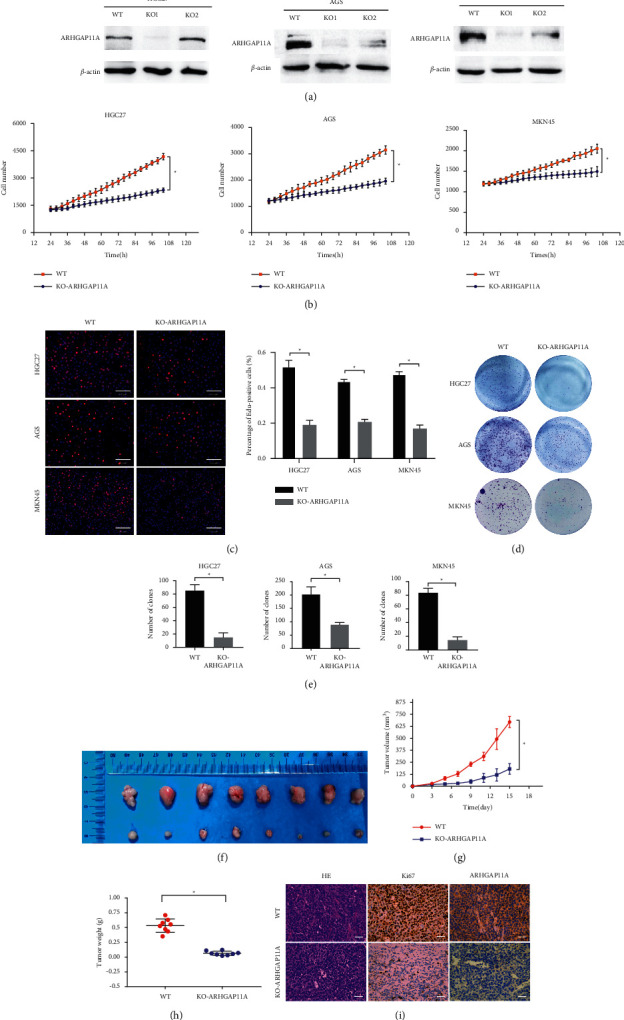
The effect of ARHGAP11A on the proliferation of gastric cancer cells in vivo and in vitro. (a) Western blot verified the ARHGAP11A knockout efficiency. (b) Through continuous observation and counting of gastric cancer cells. It was found that the proliferation rate of ARHGAP11A knockout cells was significantly reduced. (c) Cell proliferation ability was evaluated by an EdU incorporation assay, and it was found that the cell proliferation ability of gastric cancer cells with ARHGAP11A knockout was reduced. Scale bar: 100 *μ*m. ((d) and (e)) Colony formation experiments showed that the proliferation ability of ARHGAP11A knockout cells was reduced. (f) Subcutaneous tumor growth in nude mice in the control group and ARHGAP11A knockout group. (g) Tumor volumes were measured every other day. (h) Tumor weights in the two groups. ^*∗*^*p* < 0.05. (i) Representative images of tumor HE staining and Ki-67 and ARHGAP11A immunohistochemistry staining. Scale bar: 200 *μ*m.

AGS, MKN45, and HGC27 gastric cancer cells were transduced with lentiviral vectors. After puromycin screening, Western blot was performed to verify the knockout efficiency of the two targets KO1 and KO2 of ARHGAP11A. The results showed that the knockout efficiency of KO1 was obvious, while the target of KO2 was invalid (Figure 2(a)). After successfully constructing ARHGAP11A knockout cell lines in AGS, MKN45, and HGC27 cells using the KO1 target, we studied the effect of this gene on the proliferation of gastric cancer cells. The High Content Analysis System was used to continuously observe and count the cells of these three lines in a 96-well plate, and the results showed that cell proliferation slowed down after ARHGAP11A knockout (Figure 2(b)). Consistent with this finding, the results of colony formation assays showed that the number of colonies formed decreased after ARHGAP11A knockout (Figure 2(c)). In addition, the proliferation ability of gastric cancer cells was evaluated by an EdU incorporation assay, and it was found that the proliferation of gastric cancer cells slowed down after ARHGAP11A knockout (Figures 2(d) and 2(e)). Thus, we concluded that ARHGAP11A can promote the proliferation of gastric cancer cells.

To further confirm the influence of the change in the ARHGAP11A expression level on tumor growth in vivo, we established a subcutaneous xenograft model in nude mice by subcutaneous injection of ARHGAP11A knockout and negative control MKN45 cells. After 4 weeks of culture, it was found that the tumors volumes and weights in the ARHGAP11A knockout group were significantly lower than those in the control group (Figures 2(f)–2(h)), which was consistent with the results of the in vitro cell proliferation and EdU incorporation assays. Moreover, the expressions of ARHGAP11A and Ki-67 in tumor tissues were detected by immunohistochemistry. Tumors derived from ARHGAP11A knockout cells exhibited much weaker staining of Ki-67 and ARHGAP11A (Figure 2(i)). Therefore, we inferred that ARHGAP11A is closely related to the progression of gastric cancer.

### 3.3. ARHGAP11A Affects the Migration and Invasion Ability of Gastric Cancer Cells and Regulates Stress Fibers

**Figure 3:**
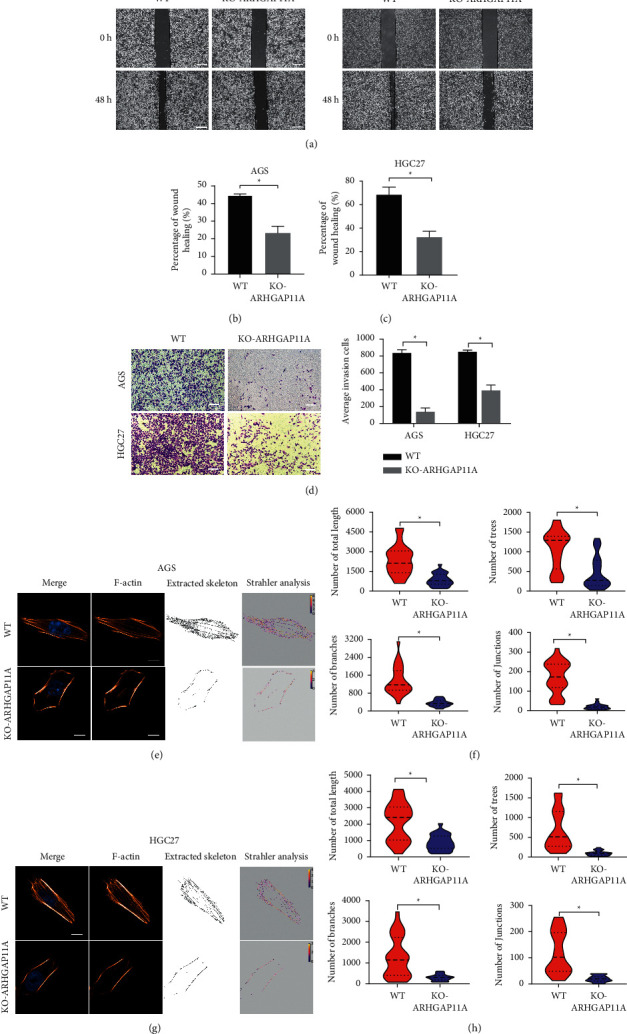
ARHGAP11A promotes the invasion and migration of gastric cancer cells and affects the formation of stress fibers. (a, c) Wound-healing assay showed that ARHGAP11A promotes gastric cancer cell migration. Scale bar: 200 *μ*m. (d) The Transwell assay showed that ARHGAP11A promotes gastric cancer cell invasion. Scale bar: 200 *μ*m. (e) Strahler analysis of stress fibers in AGS cells. Stress fiber formation was inhibited in the ARHGAP11A knockout group. Scale bar: 50 *μ*m. (f) Statistical analysis of the total length and the numbers of trees, branches, and junctions of stress fiber in AGS cells. (g) Strahler analysis of stress fiber in HGC27 cells. Scale bar: 50 *μ*m. (h) Statistical analysis of the total length, as well as the numbers of trees, branches, and junctions of the stress fiber in HGC27 cells. ^*∗*^*p* < 0.05.

To investigate the effect of ARHGAP11A expression on the migration and invasion abilities of gastric cancer cells, wound-healing assays and Transwell invasion assays were used in this study. The results of the cell wound-healing assays showed that, compared with the control group, the ARHGAP11A knockout group had a poorer ability to repair the intercellular wound (Figures 3(a)–3(c)), indicating that ARHGAP11A has an effect on the migration ability of gastric cancer cells. The results of the Transwell invasion assay showed that the number of cells passing through the Matrigel-coated membrane in the ARHGAP11A knockout group was significantly reduced compared with that in the control group (Figure 3(d)). These findings show that ARHGAP11A is involved in the invasion of gastric cancer cells. Based on the above in vitro experimental results, it was suggested that ARHGAP11A plays a role as a protooncogene in the occurrence and development of gastric cancer, promotes tumor cell invasion and migration, and leads to malignant tumor progression.

Cell migration is powered by the continuous contraction of stress fibers and the growth and extension of actin filaments; thus, we further studied whether ARHGAP11A can induce stress fiber changes in gastric cancer cells. The results showed that the number of stress fibers in the ARHGAP11A knockout group was significantly reduced and that the stress fibers were slender (Figures 3(e) and 3(g)). Strahler analysis showed that, compared with the control group, the number of stress fiber branches, the length of the stress fibers, and the degree of branching complexity were decreased significantly in the ARHGAP11A knockout group compared with the control group (Figures 3(f) and 3(h)), indicating that ARHGAP11A promotes cell migration by regulating stress fibers formation.

### 3.4. ARHGAP11A Interacts with TPM1 in Gastric Cancer

**Figure 4:**
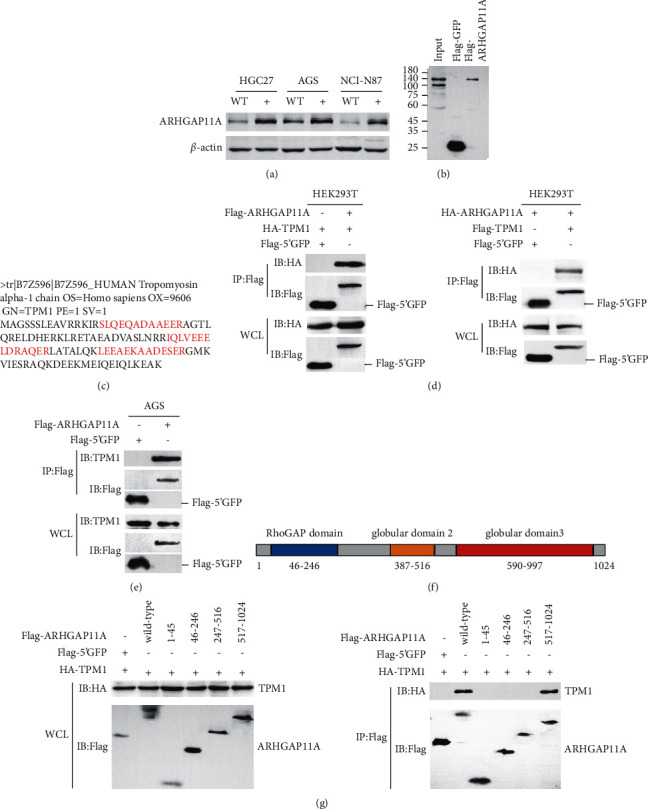
ARHGAP11A interacts with TPM1 in gastric cancer cells. (a) Western blot analysis verified the overexpression efficiency of ARHGAP11A in gastric cancer cells. (b) Western blot detection after IP. (c) Matching peptides in ARHGAP11A and TPM1. (d) Left: Flag-tagged ARHGAP11A and HA-tagged TPM1 plasmids were co-transfected into HEK293T cells for 36 h followed by cell lysate preparation and IP assay with anti-Flag beads followed by immunoblotting with indicated antibodies. Right: HA-tagged ARHGAP11A and Flag-tagged TPM1 plasmids were cotransfected into HEK293T cells for 36 h followed by cell lysate preparation and IP assay with anti-Flag beads followed by immunoblotting with indicated antibodies. IP: immunoprecipitates, WCL: whole-cell lysates. (e) The interaction of ARHGAP11A and TPM1 was tested in AGS cells. AGS cells overexpressing Flag-ARHGAP11A were lysed with cell lysate, followed by IP assay with anti-Flag beads followed by immunoblotting with indicated antibodies. (f) ARHGAP11A domain. (g) Analysis of the domain involved in the interaction between ARHGAP11A and TPM1. HEK293T cells were transiently cotransfected with plasmids expressing Flag-tagged of indicated ARHGAP11A mutant plasmids and HA-tagged TPM1 plasmids, followed by cell lysate preparation and IP assay with anti-Flag beads followed by immunoblotting with indicated antibodies. IP: immunoprecipitates, WCL: whole-cell lysates.

ARHGAP11A overexpression cell lines were generated with AGS, HGC27, and NCI-N87 cells, and the overexpression efficiency was verified by Western blot analysis (Figure 4(a)). In AGS cells, protein-protein interactions were identified by mass spectrometry combined with bioinformatics analysis, and it was found that the actin-binding protein TPM1 interacted with ARHGAP11A (Figures 4(b) and 4(c)). TPM1, a member of the tropomyosin family, is a cytoskeletal protein that binds to actin in various cells [11]. TPM1 has increased functional complexity in nonmuscle cells, and its main role is to stabilize the cytoskeleton [12]. The interaction between ARHGAP11A and TPM1 was confirmed in both HEK293T and AGS cells by coimmunoprecipitation (Figures 4(d) and 4(e)).

Next, to identify the domain via which ARHGAP11A interacts with TPM1, according to the predicted domains of ARHGAP11A (Figure 4(f)) reported in the literature [13], truncation mutants of ARHGAP11A 1–45, 46–246, 247–516, and 517–1024 were constructed, and the designed primer sequences are shown in Table S2. An anti-Flag antibody was used as “bait” for immunoprecipitation of TPM1, and the results showed that the domain of ARHGAP11A involved in its interaction with TPM1 was 517–1024 (Figure 4(g)).

### 3.5. TPM1 Plays a Central Role in the Malignant Transformation of Gastric Cancer Induced by ARHGAP11A

**Figure 5:**
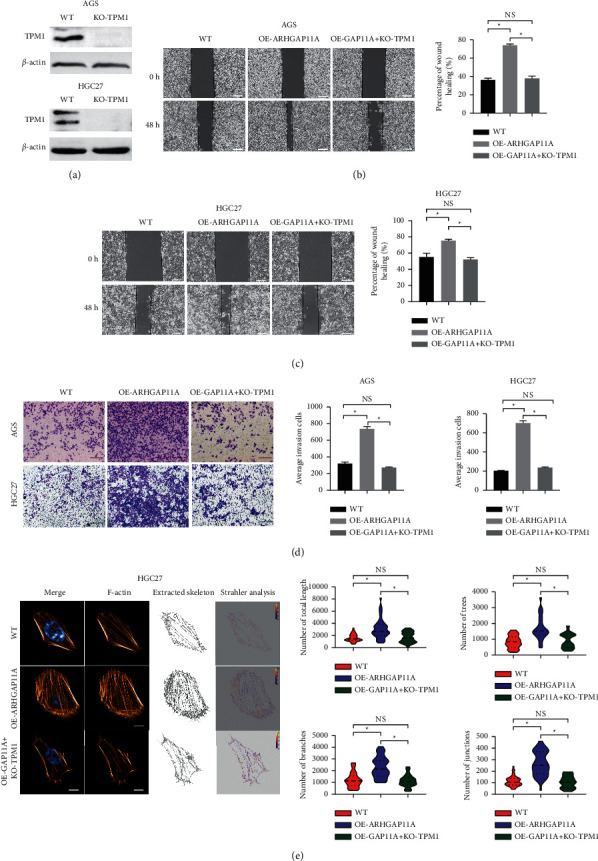
TPM1 is essential for cell invasion and migration induced by ARHGAP11A. (a) The overexpression efficiency of TPM1 was verified by Western blot analysis. (b) The cell migration ability of WT, OE-ARHGAP11A, and OE-ARHGAP11A + KO-TPM1 cells was evaluated by a wound-healing assay. (c) A Transwell assay was used to evaluate the invasion ability of WT, OE-ARHGAP11A, and OE-GAP11A + KO-TPM1 cells. Scale bar: 200 *μ*m. (d) Detection of stress fibers in HGC27 gastric cancer cells. (e) Statistical analysis of the total length, as well as the number of trees, branches, and junctions of stress fibers in HGC27 cells. Scale bar: 50 *μ*m. ^*∗*^*p* < 0.05.

To further confirm that ARHGAP11A promotes the invasion and migration of gastric cancer through TPM1, TPM1 was knocked out in AGS and HGC27 cells with stable and high expression of ARHGAP11A (OE-GAP11A + KO-TPM1). Cells were divided into three groups: WT, OE-ARHGAP11A, and OE-GAP11A + KO-TPM1. We used wound-healing assays to evaluate the cell migration ability, and the results showed that ARHGAP11A overexpression promoted the migration ability of gastric cancer cells compared with that of the WT cells (Figure 5(a)). The migration ability of OE-GAP11A + KO-TPM1 cells was significantly decreased compared with that of ARHGAP11A overexpressing cells (Figures 5(b) and 5(c)). The invasion ability of gastric cancer cells was further evaluated by Transwell assays, and similar conclusions were drawn. The invasion ability of OE-GAP11A + KO-TPM1 cells was significantly lower than that of the ARHGAP11A overexpressing cells (Figure 5(d)). The above findings show that ARHGAP11A's ability to promote migration and invasion depends on TPM1.

Immunofluorescence staining was performed to detect the stress fibers labeled with Rhodamine-phalloidin in the WT, OE-ARHGAP11A, and OE-GAP11A + KO-TPM1 groups, and the results showed that the stress fibers exhibited fluorescence in a filamentous pattern. Compared with those in the WT group, the number and length of stress fibers in the OE-ARHGAP11A group was significantly increased, and the stress fibers were thicker. Strahler analysis showed that the total length of the stress fibers and the numbers of trees, branches, and junctions in the OE-GAP11A group were significantly higher than those in the WT group. Compared with the OE-GAP11A group, the OE-GAP11A + KO-TPM1 group had a simpler microfilament skeleton structure and a reduced number of stress fibers (Figure 5(e) and Supplementary Figure S2), suggesting that the function of ARHGAP11A in regulating stress fiber formation is dependent on TPM1.

In summary, TPM1 is an interacting protein of ARHGAP11A that plays a key role in the ARHGAP11A-induced malignant progression of gastric cancer.

## 4. Discussion

ARHGAP11A is a member of the Rho GTPase-activating protein family, but its role in gastric cancer has not been elucidated, and the related mechanisms have not been thoroughly explored. In this study, we found that the expression of ARHGAP11A is positively correlated with a low degree of tumor differentiation and low survival rate of human gastric cancer patients. Further in vivo and in vitro analyses confirmed that the role of ARHGAP11A in gastric cancer cells is to promote cell proliferation, migration, and invasion. The above data indicate that ARHGAP11A plays an important role in the malignant progression of gastric cancer.

The cytoskeleton mainly consists of microfilaments, microtubules, and intermediate filaments, and the main component of microfilaments is actin [14]. In cells cultured in vitro, there are a large number of stable and parallel microfilament structures on the inner side of the plasma membrane close to focal adhesion; these structures are called stress fibers and are composed of actin, myosin, tropomyosin, and so forth [15]. The contractile force produced by the relative movement of actin and myosin is the main driver of cell migration. Studies have shown that, in addition to contributing to cell migration and morphogenesis, stress fibers also contribute to adhesion, mechanical conduction, endothelial barrier integrity, and myofibril assembly [16]. Our study showed that the number of cellular stress fibers was significantly reduced after ARHGAP11A knockout and that the invasion and migration abilities of the knockout cells were significantly decreased, suggesting that ARHGAP11A may be involved in the malignant transformation of cells by affecting the mechanism of stress fiber polymerization and depolymerization.

TPM1 is a member of the tropomyosin family and is a cytoskeletal protein that binds to actin in a variety of cells [17]. TPM1 plays a role in the troponin complex, which regulates the contraction of muscle cells in a calcium-dependent manner, while its functional complexity is increased in nonmuscle cells, in which it mainly stabilizes the cytoskeleton [12]. It is reported that TPM1 is a new predictive biomarker for gastric cancer diagnosis and prognosis [17]. Our study showed that ARHGAP11A can interact with TPM1 in gastric cancer cells, thereby promoting gastric cancer progression by affecting the formation and stability of the actin filaments.

## 5. Conclusion

In summary, our research reveals the promoting role of ARHGAP11A in the malignant development of gastric cancer and identifies the mechanism by which ARHGAP11A plays its role. As an oncogene in gastric cancer, ARHGAP11A is dependent on TPM1 to regulate cell stress fiber formation and stability and promote gastric cancer cell proliferation, invasion, and migration, thus promoting gastric cancer progression.

## Figures and Tables

**Table 1:** Primers for qRT-PCR analysis.

Gene	Primer (forward: 5′–3′)	Primer (reverse: 5′–3′)
ARHGAP11A	ATATTGGGCGTGTACCAGATTTT	CAATGTACGCTTAGCATTTGGTG
GAPDH	GCACCGTCAAGGCTGAGAAC	TGGTGAAGACGCCAGTGGA

**Table 2:** Clinical correlation of ARHGAP11A expression in gastric cancer.

Clinical characteristic	Variable	*N*	ARHGAP11A expression		*χ*2	*p* value
			Low	High		
*Gender*	Male	331	110	221	0.037	0.908
	Female	111	38	73		

*Age (y)*	≤50	113	37	76	0.037	0.908
	>50	329	111	218		

*Tumor size (cm* ^*3*^)	≤5	317	112	205	1.717	0.219
	>5	125	36	89		

*Tumor differentiation*	Well	28	8	20	9.676	0.008
	Moderate	238	95	143		
	Poor	176	45	131		

*Clinical stage*	I	79	26	53	1.839	0.606
	II	166	52	114		
	III	177	65	112		
	IV	20	5	15		

*T classification*	T1	51	21	30	2.914	0.405
	T2	79	30	49		
	T3	186	57	129		
	T4	126	40	86		

*N classification*	N0	160	57	103	5.699	0.127
	N1	89	37	52		
	N2	71	21	50		
	N3	122	33	89		

*M classification*	M0	428	146	282	2.393	0.156
	M1	14	2	12		

*Nerve and vascular invasion*	Yes	372	124	248	0.024	0.891
	NO	70	24	46		

## Data Availability

The data used to support the findings of this study are included within the article.

## Supplementary Materials

Supplementary Materials Table S1: primer sequences of the stable transfection plasmid and transient transfection plasmid for ARHGAP11A. Table S2: primer sequences of truncation mutants for ARHGAP11A. Figure S1: expression of ARHGAP11A in various cancers of the digestive system. ^*∗*^*p* < 0.05. Figure S2: stress fibers in the WT, OE-ARHGAP11A, and OE-GAP11A + KO-TPM1 groups of AGS gastric cancer cells. (A) Representative images of stress fibers in each group. Scale bars: 50 *μ*m. (B) Statistical analysis of the total length, and the numbers of trees, branches, and junctions of stress fibers in AGS cells. ^*∗*^*p* < 0.05 .
