# Supplementary material for: ARHGAP11A Promotes the Malignant Progression of Gastric Cancer by Regulating the Stability of Actin Filaments through TPM1
Source: J Oncol. 2021 Dec 6;2021:4146910. doi: 10.1155/2021/4146910 (PMC8668285; doi:10.1155/2021/4146910)
Supplement: Supplementary Materials — Table S1: primer sequences of the stable transfection plasmid and transient transfection plasmid for ARHGAP11A. Table S2: primer sequences of truncation mutants for ARHGAP11A. Figure S1: expression of ARHGAP11A in various cancers of the digestive system. ∗p < 0.05. Figure S2: stress fibers in the WT, OE-ARHGAP11A, and OE-GAP11A + KO-TPM1 groups of AGS gastric cancer cells. (A) Representative images of stress fibers in each group. Scale bars: 50 μm. (B) Statistical analysis of the total length, and the numbers of trees, branches, and junctions of stress fibers in AGS cells. ∗p < 0.05 . [file 4146910.f1.zip › 4146910.f1/Supplementary table file 1 (1).docx]

Table S1 Primer sequences of the stable transfection plasmid and transient transfection plasmid for ARHGAP11A.

| Plasmid | Primer sequences |
| --- | --- |
| GAP11A-P-F-2-520-FW | TACAAGGACGATGACGATAAGTGGGATCAGAGGCTGGTGAGG |
| GAP11A-P-F-2-520-RV | GCTTCCCGAATTCTAGACCCGGGATCCAGACATCCGGTAATTTGTTCC |
| GAP11A-P-F-2-1203-FW | GGAACAAATTACCGGATGTCTTGGACAGGACCTAATAATTCA |
| GAP11A-P-F-2-1203-RV | GCTTCCCGAATTCTAGACCCGCTACAAATCTACAGGTTTACTTGT |
| GAP11A-L-F-2-520-FW | TACAAGGACGATGACGATAAGT GGGATCAGAGGCTGGTGAGG |
| GAP11A-L-F-2-520-RV | GTCCATGAATTCTAGACCCTAGGATCCAGACATCCGGTAATTTGTTCC |
| GAP11A-L-F-2-1203-FW | GGAACAAATTACCGGATGTCT TGGACAGGACCTAATAATTCA |
| GAP11A-L-F-2-1203-RV | GTCCATGAATTCTAGACCCTA CTA CAAATCTACAGGTTTACTTGT |
| P-H-GAP11A-2-250-FW | CCAGATTACGCTGCTCAGCGCTGGGATCAGAGGCTGGTGAGG |
| P-H-GAP11A-2-250-RV | CCAAGCTTCCCGAATTCTAGACCCGGGAGACATCCGGTAATTTGTTCC |
| P-H-GAP11A-2-1023-FW | GGAACAAATTACCGGATGTCTTGGACAGGACCTAATAATTCA |
| P-H-GAP11A-2-1023-RV | GCTTCCCGAATTCTAGACCCGTCACAAGACAAGGCACCCAGA |

Note: GAP11A-P-F means transient transfection plasmid PRK5-Flag-ARHGAP11A, GAP11A-L-F means stable transfection plasmid Lenti-CMV-Flag-ARHGAP11A, P-H-GAP11A means transient transfection plasmid PRK5-HA-ARHGAP11A.
