# Supplementary figures and images for: ARHGAP11A Promotes the Malignant Progression of Gastric Cancer by Regulating the Stability of Actin Filaments through TPM1
Source: J Oncol. 2021 Dec 6;2021:4146910. doi: 10.1155/2021/4146910 (PMC8668285; doi:10.1155/2021/4146910)

Supplementary Figure 1


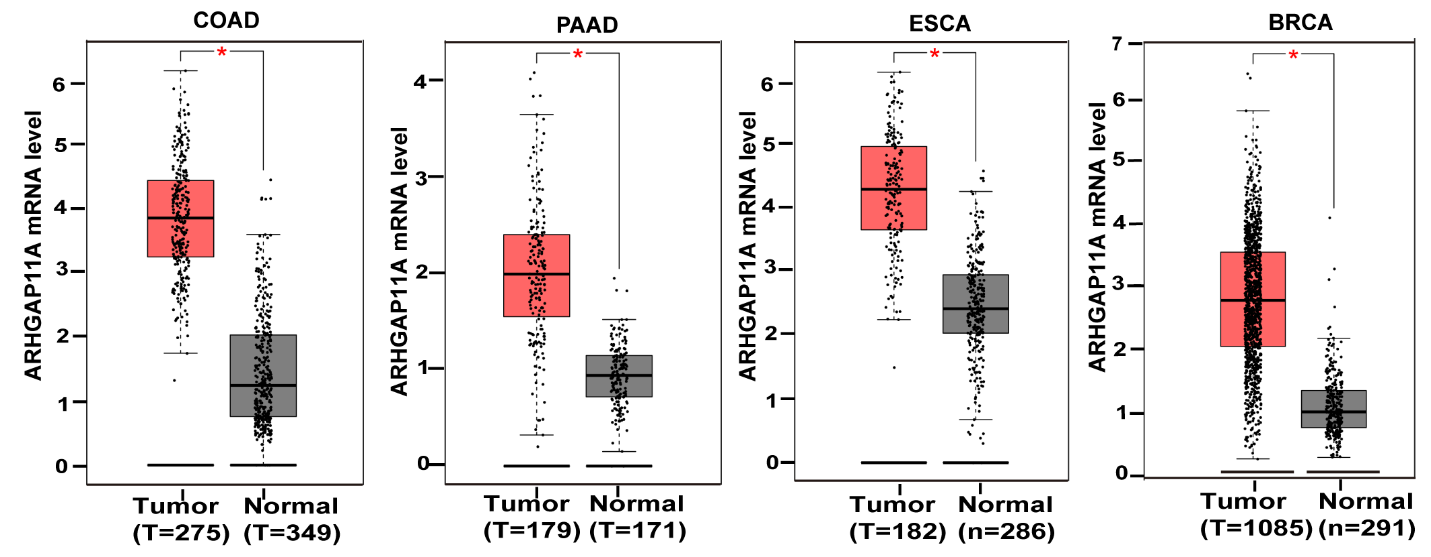


Supplementary figure 2


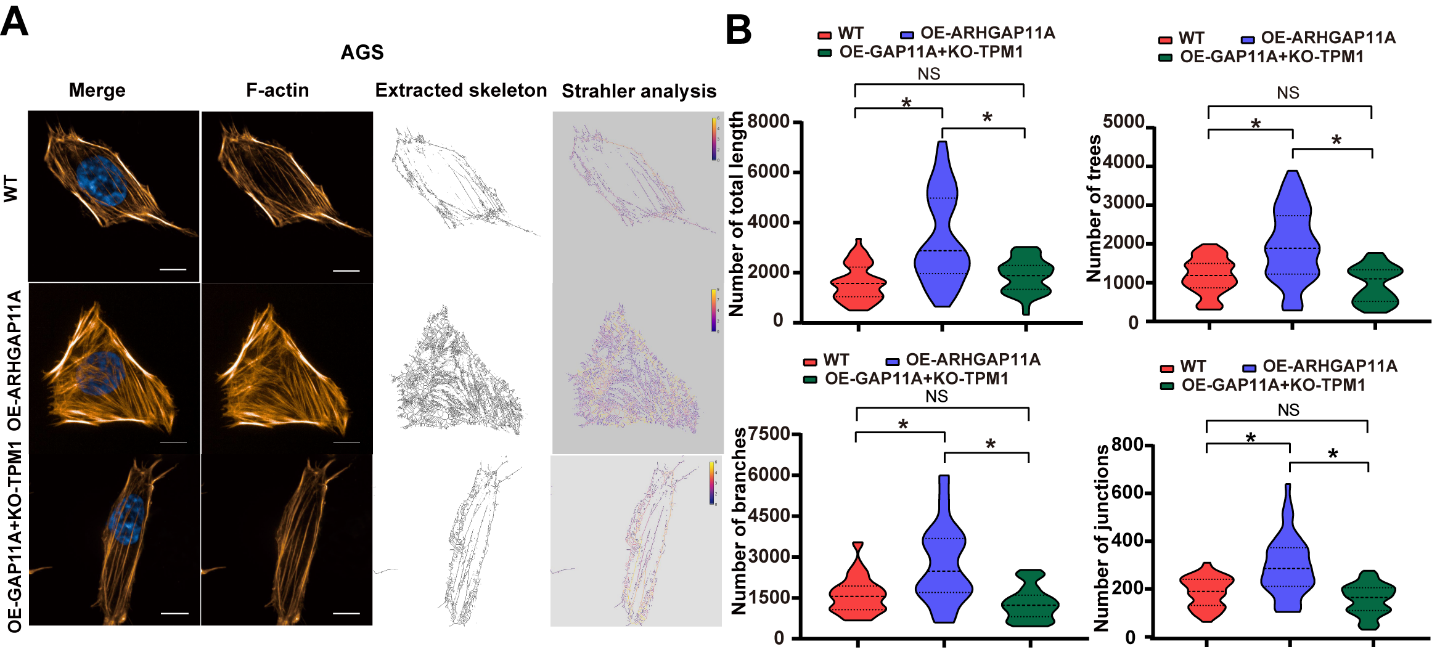

Supplement: Supplementary Materials — Table S1: primer sequences of the stable transfection plasmid and transient transfection plasmid for ARHGAP11A. Table S2: primer sequences of truncation mutants for ARHGAP11A. Figure S1: expression of ARHGAP11A in various cancers of the digestive system. ∗p < 0.05. Figure S2: stress fibers in the WT, OE-ARHGAP11A, and OE-GAP11A + KO-TPM1 groups of AGS gastric cancer cells. (A) Representative images of stress fibers in each group. Scale bars: 50 μm. (B) Statistical analysis of the total length, and the numbers of trees, branches, and junctions of stress fibers in AGS cells. ∗p < 0.05 . [file 4146910.f1.zip › 4146910.f1/Supplementary Figures.docx]
